# Supplementary material for: Modeling glioblastoma heterogeneity as a dynamic network of cell states
Source: Mol Syst Biol. 2021 Sep 16;17(9):e10105. doi: 10.15252/msb.202010105 (PMC8444284; doi:10.15252/msb.202010105)
Supplement: Supplementary file 6 — Source Data for Figure 5 [file MSB-17-e10105-s004.zip › Figure5A_sourcedata/GSEA_3017/hallmarks_stateC.GseaPreranked.1621934520804/HALLMARK_INFLAMMATORY_RESPONSE.html]

Details for gene set HALLMARK\_INFLAMMATORY\_RESPONSE[GSEA]

|  || Dataset | state13017 |
| Phenotype | NoPhenotypeAvailable |
| Upregulated in class | na\_neg |
| GeneSet | HALLMARK\_INFLAMMATORY\_RESPONSE |
| Enrichment Score (ES) | -0.5051903 |
| Normalized Enrichment Score (NES) | -2.1438057 |
| Nominal p-value | 0.0 |
| FDR q-value | 0.0044870973 |
| FWER p-Value | 0.017 |
Table: GSEA Results Summary

  

Fig 1: Enrichment plot: HALLMARK\_INFLAMMATORY\_RESPONSE      
 Profile of the Running ES Score & Positions of GeneSet Members on the Rank Ordered List

  

| PROBE | GENE SYMBOL | GENE\_TITLE | RANK IN GENE LIST | RANK METRIC SCORE | RUNNING ES | CORE ENRICHMENT || 1 | OSMR |  |  | 292 | -0.274 | -0.4690 | Yes |
| 2 | NAMPT |  |  | 306 | -0.280 | -0.4544 | Yes |
| 3 | CSF1 |  |  | 320 | -0.285 | -0.4392 | Yes |
| 4 | SERPINE1 |  |  | 323 | -0.286 | -0.4049 | Yes |
| 5 | AHR |  |  | 324 | -0.286 | -0.3670 | Yes |
| 6 | CHST2 |  |  | 350 | -0.297 | -0.3709 | Yes |
| 7 | TNFAIP6 |  |  | 359 | -0.301 | -0.3449 | Yes |
| 8 | PDPN |  |  | 372 | -0.307 | -0.3250 | Yes |
| 9 | TIMP1 |  |  | 399 | -0.320 | -0.3275 | Yes |
| 10 | SLC4A4 |  |  | 415 | -0.332 | -0.3096 | Yes |
| 11 | LDLR |  |  | 419 | -0.334 | -0.2706 | Yes |
| 12 | MYC |  |  | 421 | -0.336 | -0.2278 | Yes |
| 13 | KLF6 |  |  | 429 | -0.339 | -0.1951 | Yes |
| 14 | PTPRE |  |  | 469 | -0.372 | -0.2133 | Yes |
| 15 | HBEGF |  |  | 480 | -0.386 | -0.1794 | Yes |
| 16 | F3 |  |  | 505 | -0.432 | -0.1637 | Yes |
| 17 | EMP3 |  |  | 568 | -0.580 | -0.1941 | Yes |
| 18 | HAS2 |  |  | 587 | -0.740 | -0.1273 | Yes |
| 19 | CCL2 |  |  | 595 | -1.066 | 0.0017 | Yes |
Table: GSEA details [plain text format]

  

Fig 2: HALLMARK\_INFLAMMATORY\_RESPONSE: Random ES distribution      
 Gene set null distribution of ES for **HALLMARK\_INFLAMMATORY\_RESPONSE**
